# Supplementary material for: Reversible silencing of lumbar spinal interneurons unmasks a task-specific network for securing hindlimb alternation
Source: Nat Commun. 2017 Dec 6;8:1963. doi: 10.1038/s41467-017-02033-x (PMC5719045; doi:10.1038/s41467-017-02033-x)
Supplement: Supplementary file 1 — Supplementary Information [file 41467_2017_2033_MOESM1_ESM.pdf]

Supplementary Figure 1.

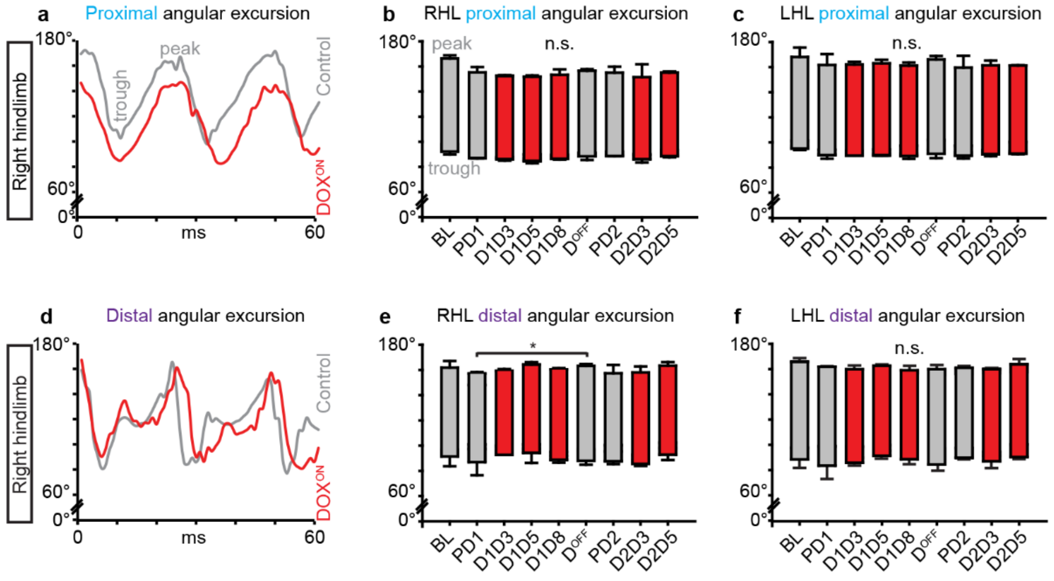

Supplementary Figure 1. Silencing L2-L5 interneurons does not affect hindlimb range-of-motion. Representative traces of the proximal (**a**) and distal (**d**) hindlimb angle excursions (from peak to trough) at Control (gray) and DOX^{ON} time points (red), respectively (right hindlimb shown). Silencing L2-L5 interneurons did not affect the peak-to-trough excursion of the right (**b**) or left (**c**) proximal hindlimb angle. Similarly, the conditional silencing did not alter the excursion of the distal hindlimb angle for the right (**e**) or left hindlimb (**f**), respectively. There was a slight, but significant increase in the angular excursion at DOX^{OFF} as compared to Pre-DOX1 (*$p<0.05$; mixed model ANOVA and Bonferroni *post hoc* t-test). Quantitative data are the mean ± S.D. (N=6).

Supplementary Figure 2.

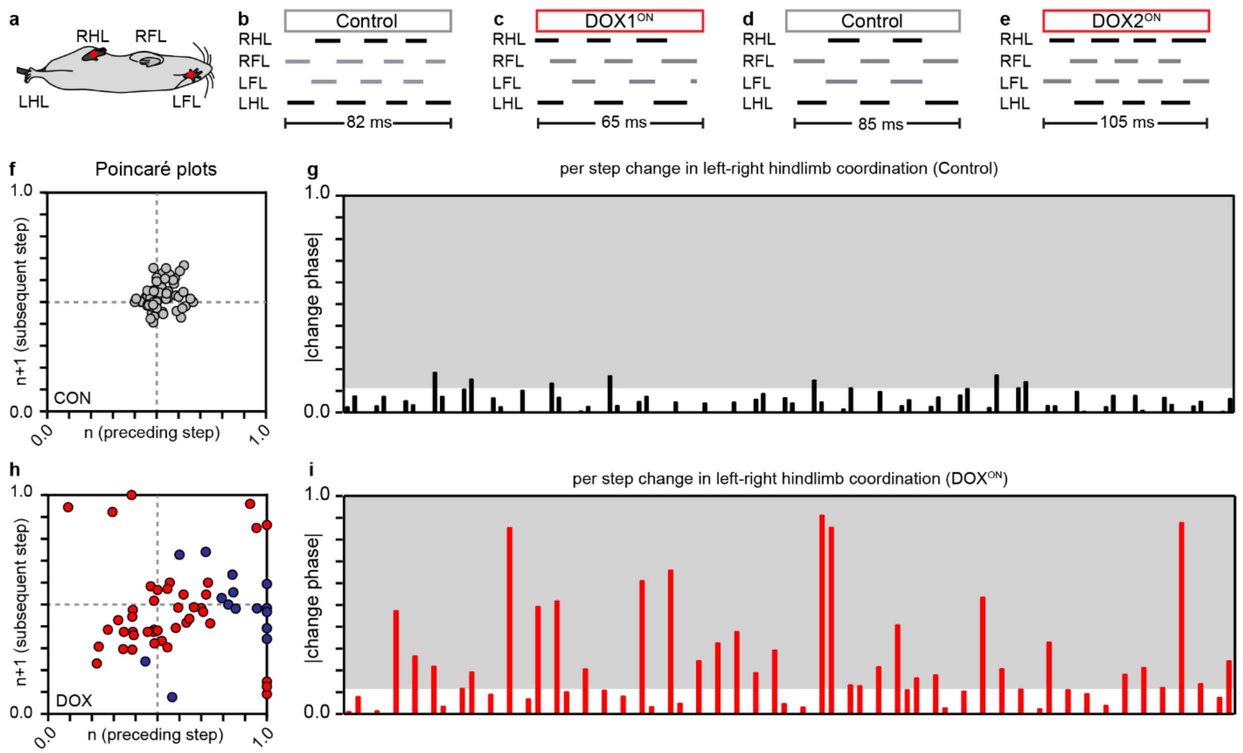

Supplementary Figure 2. Silencing L2-L5 interneurons disrupts left-right hindlimb coordination on a step-by-step basis. (**a**) Ventral recordings of paw contacts were used to generate swing-stance graphs and gait analyses (LFL: left forelimb; RFL: right forelimb; LHL: left hindlimb; RHL: right hindlimb). (**b-e**) Swing-stance graphs from Control and DOX^{ON} time points, respectively, with the duration of the overall locomotor bout shown below. Each line denotes paw contact with the ground (stance phase) while the intervening space between each line represents the swing phase, when the paw is in the air. A subset of the data shown in Figure 5f was selected for expanded graphical representation of per-step changes in left-right hindlimb coordination (**g,i**, expanded for clarity; shaded region denotes changes beyond control variability) as well as Poincaré plot generation (**f,h**), which illustrate left-right hindlimb coordination across successive step cycles (x-axis, preceding step *n*; y-axis, subsequent step *n+1*). N=95 and 96 per-step changes were sampled from Control and DOX^{ON} time points, respectively.

Supplementary Figure 3.

a Average locomotor speed

Time point	cm/s
Baseline	48.83±4.16
Pre-DOX1	50.39±4.24
DOX1^{ON}-D3	57.98±4.92
DOX1^{ON}-D5	78.20±9.46*
DOX1^{ON}-D8	85.18±12.76*
DOX^{OFF}	64.81±12.73
Pre-DOX2	58.74±10.90
DOX2^{ON}-D3	76.28±7.73**
DOX2^{ON}-D5	76.68±5.89**
Control (min-max)	48.83±4.16 - 64.81±12.73
DOX^{ON} (min-max)	57.98±4.92 - 85.18±12.76

b Hindlimb phase verus speed

Time point	r value	p value
Baseline	-0.023	0.965
Pre-DOX1	-0.677	0.140
DOX1^{ON}-D3	0.455	0.365
DOX1^{ON}-D5	-0.468	0.349
DOX1^{ON}-D8	-0.082	0.878
DOX^{OFF}	0.613	0.196
Pre-DOX2	-0.367	0.475
DOX2^{ON}-D3	0.001	0.999
DOX2^{ON}-D5	0.097	0.855
All >2 S.D.	0.005	0.980

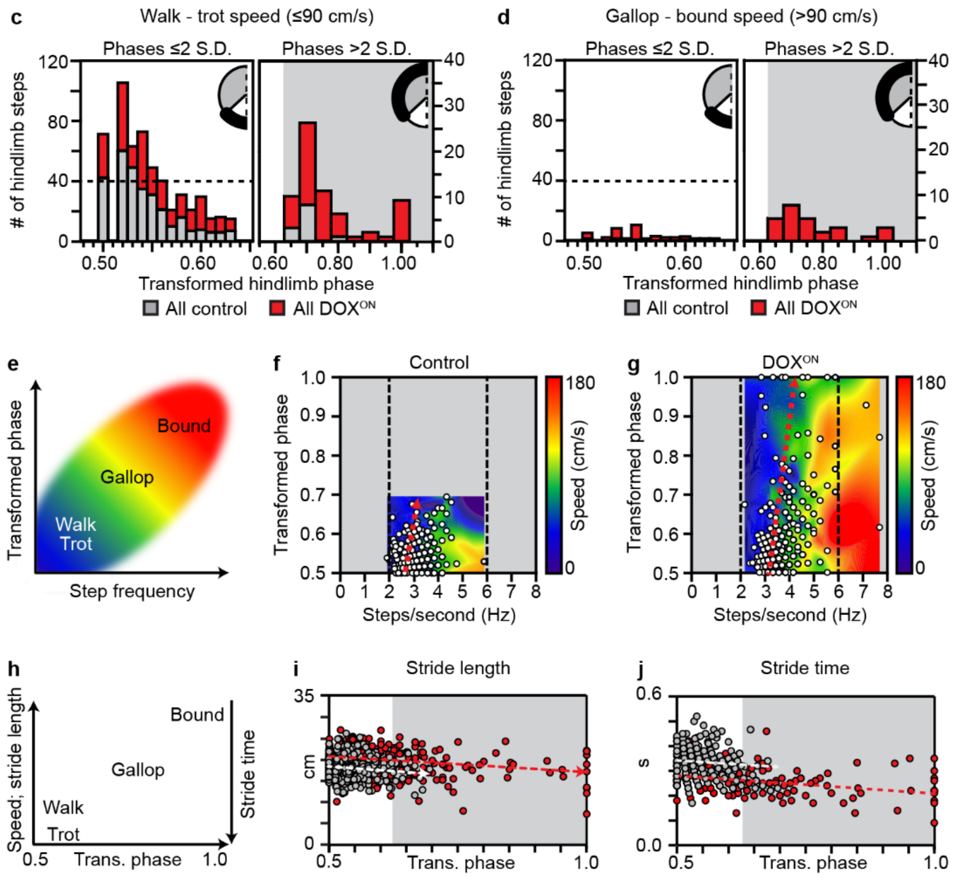

Supplementary Figure 3. Silencing-induced changes in hindlimb coordination did not correlate with speed or gait-related indices. (**a**) Overall locomotor speed across time points (group mean±S.D.). The speed was significantly enhanced during DOX1^{ON}-D5 and DOX1^{ON}-D8 as compared to Pre-DOX1 (* p≤0.5, repeated measures ANOVA; Tukey's *post hoc* honest significant difference t-test) as well as during DOX2^{ON}-D3 and DOX2^{ON}-D5 as compared to Pre-DOX2 (**p≤0.01). No significant different differences were detected when comparing to Baseline and DOX^{OFF}. The overall speed range

(minimum-maximum) for the group at Control and DOX^{ON} time points, respectively, is listed below. (**b**) Summary of Pearson correlations between averaged phase vs speed, per time point. (**c**) Left panel shows the frequency distribution of hindlimb coordination values within the control variability (right inset; 0.50-0.63) that occurred at ≤90 centimeters/second, a locomotor velocity where the limbs typically alternate in a walk or trot gait. Right panel shows the frequency at which phases >0.63, including synchrony at 1.0, occur at a speed where alternation usually prevails (shaded region denotes phases beyond control variability). (**d**) Frequency distribution of hindlimb phases at gallop-to-bound speeds (>90 cm/sec). (**e,h**) Inter-relationship between various gait indices. (**f,g**) Step frequency-phase relationship (white circles) mapped onto speed contour plot (see methods for detail). Silencing-induced changes in hindlimb coordination did not correlate with increased step frequency. (**i**) Similarly, changes to hindlimb coordination did not correlate with increased stride length (Control, r_S=-0.068 in dashed white line; DOX^{ON}, r_S=-0.125 in dashed red line; Spearman Rank correlation) or decreased stride time (**j**, Control r_S=-0.036; DOX^{ON} r_S=-0.338).

Supplementary Figure 4.

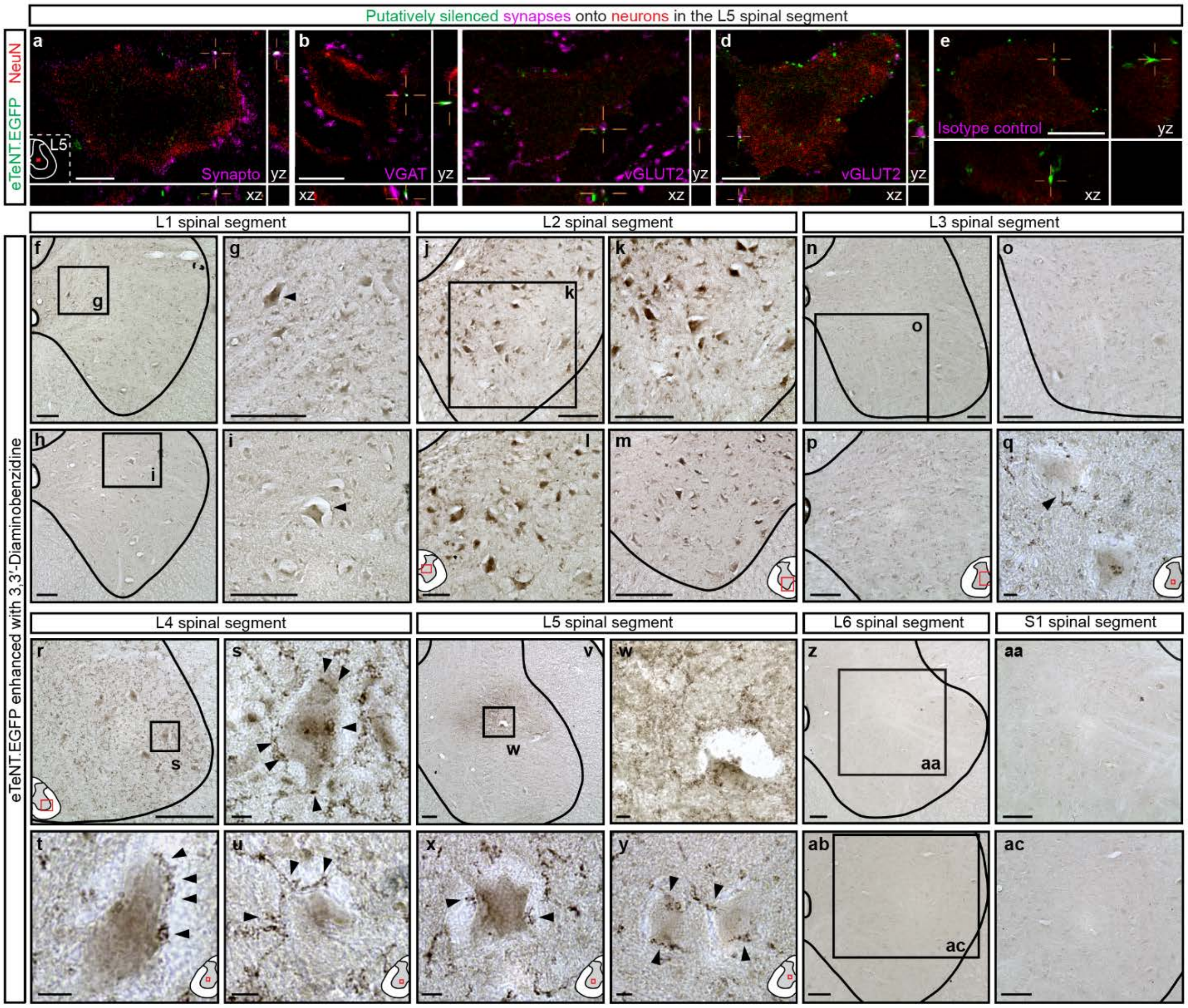

Supplementary Figure 4. Immunohistochemical interrogation of conditionally silenced L2-L5 interneurons. Cross-sections throughout the z-stack confirming co-localization of eTeNT.EGFP with synaptophysin (**a**, synapto), vesicular GABA tranporter (**b**, VGAT), or vesicular glutamate transporter 2 (**c-d**) in the xz-yz planes (white signal denoted in cross-hairs). (**e**) Isotype control. (**f-ac**) eTeNT.EGFP immunoreactivity assessed throughout the lumbosacral enlargement using 3,3’-Diaminobenzidine enhancement. (**f-i**) Few double-infected neurons were detected at L1 (black arrowheads). (**j-m**) Numerous double-infected neurons were detected throughout the intermediate gray matter at spinal L2 (black arrowheads). (**n-q**) Sparse, terminal-like structures were detected at L3 (**q**, arrowhead). No double-infected neurons were detected. (**r-u**) Terminal-like structures were detected throughout intermediate and

ventral gray matter at spinal L4 through L5 (arrowheads). (**v-y**) Cross-sections distal to lentiviral vector injection. (**z-ac**) No eTeNT.EGFP immunoreactivity was detected at L6-S1. Images acquired at 10, 20, and 40x magnifications. Scale bar=10 µm (**a-e, q, s-u, w-x, y**) or 100 µm (**f-p, r, v, z-aa, ab-ac**).

Supplementary Figure 5.

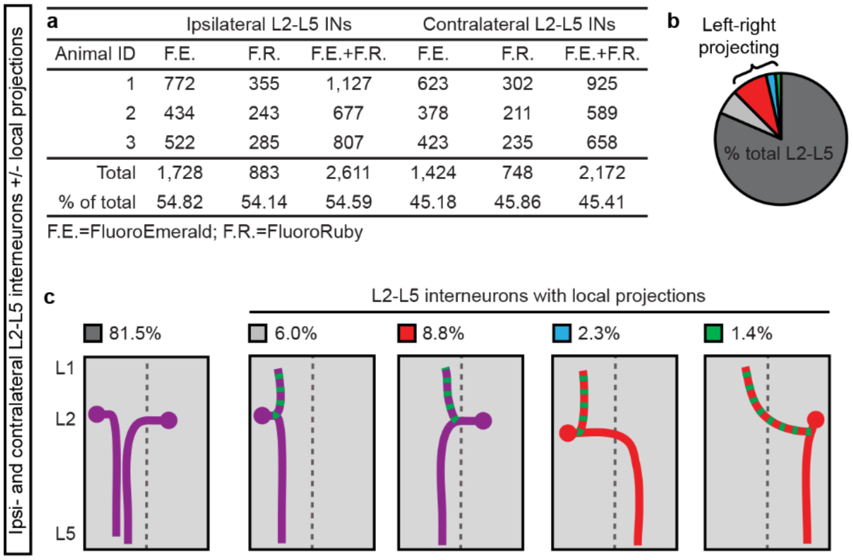

Animal ID	Ipsilateral L2-L5 INs F.E.	Ipsilateral L2-L5 INs F.R.	Ipsilateral L2-L5 INs F.E.+F.R.	Contralateral L2-L5 INs F.E.	Contralateral L2-L5 INs F.R.	Contralateral L2-L5 INs F.E.+F.R.
1	772	355	1,127	623	302	925
2	434	243	677	378	211	589
3	522	285	807	423	235	658
Total	1,728	883	2,611	1,424	748	2,172
% of total	54.82	54.14	54.59	45.18	45.86	45.41

Supplementary Figure 5. The majority of L2-L5 interneurons lack local projections in the rostral lumbar spinal cord. (**a**) Data shown are absolute cell counts and percent total of L2 interneurons with ipsilateral or contralateral projections to spinal L5 following bilateral injections of FluoroEmerald (F.E.) and FluoroRuby (F.R.). No significant difference was found between ipsi- and contralateral L2-L5 interneurons (total ipsilateral vs total contralateral: $p>0.4$; independent t-test between means of equal variance). (**b**,**c**) Summary of the L2-L5 projection patterns observed following triple tracer (CTB) injections. Of the total L2-L5 interneurons labeled at L2 following bilateral L5 injections, approximately 80% did not have local projections within one (rostral) segment of their cell body (dark gray, 81.49±2.36%). Almost 20% of the L2-L5 interneurons had projections within one segment of their cell body (18.51±2.36%). Of this proportion, approximately 12.5% had direct projections between the left and right sides of the spinal cord (**c**, red, blue, and green). Data shown represent the proportions of projection patterns observed relative to percent total L2-L5 interneurons that were labeled.

Supplementary Figure 6.

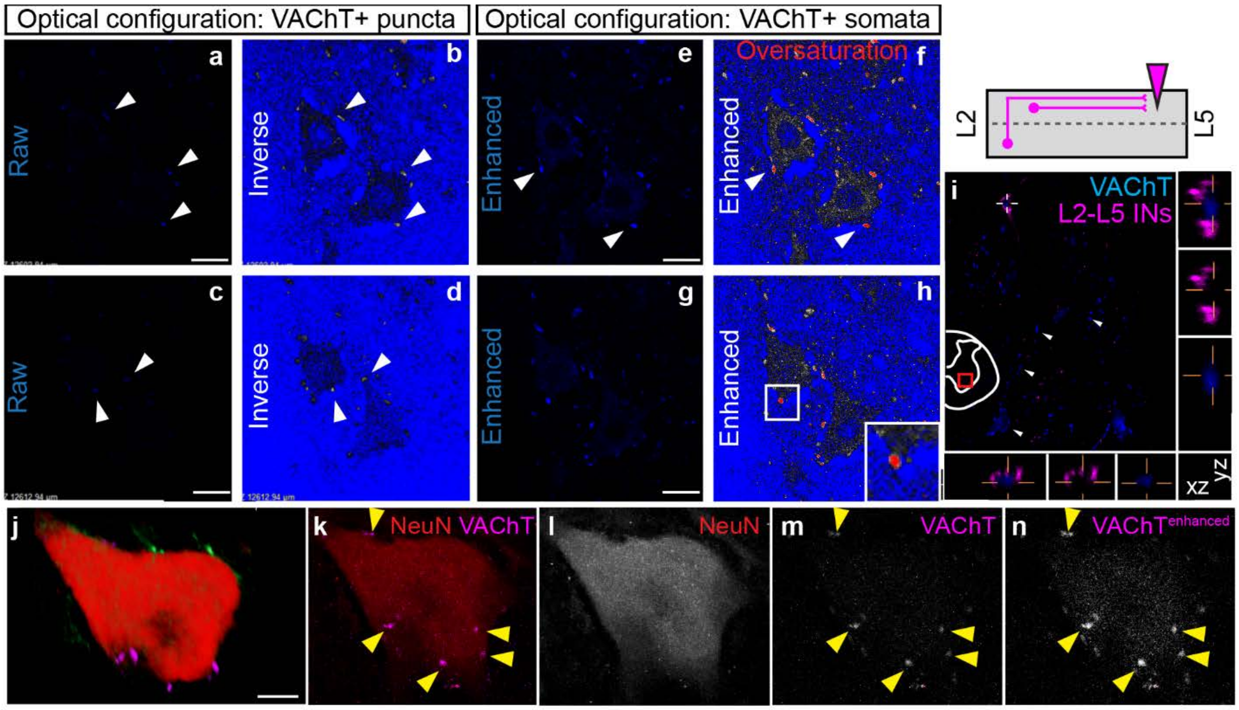

Supplementary Figure 6. **Putatively silenced synapses onto caudal lumbar motor neurons.**

(**a-d**) Optimal optical configurations for VAChT immunoreactivity in pre-synaptic puncta vs motor neuron somata (not shown: CTB-labeled motor neurons from hindlimb intramuscular injections; see "ghost" neurons in **b** and **d**). Images shown in (**a-b**) and (**c-d**) reflect VAChT-positive immunoreactivity with optimal confocal configurations set for the pre-synaptic puncta. Left panels reflect raw VAChT signal (blue). Right panels reflect the inverted image to show saturation of the fluorophore based on image acquisition settings. Ideal optical configurations for fluorophore detection yield gray-white signal (white arrowheads). Images shown in (**e-f**) and (**g-h**) reflect VAChT-positive immunoreactivity with optical configurations set for the somata. Note that when VAChT signal is detected in the somata, the pre-synaptic puncta become oversaturated (red signal, white arrowheads). Images shown reflect one optical slice (0.4 um) through a 20-30 slice z-stack captured at 100x (scale bar=20 µm). A subset of L2-L5 interneurons showed

somatic co-localization with VAChT (**i**). (**j-n**) *Post hoc* image modifications reveal VAChT-positive immunoreactivity in putative NeuN-positive motor neuron shown in Figure 7e.

Supplementary Table 1.

	Stance time		Swing time		Stride time		Stride length	
	**r value**	**p value**	**r value**	**p value**	**r value**	**p value**	**r value**	**p value**
Baseline	-0.023	0.965	0.574	0.234	0.508	0.304	0.648	0.164
Pre-DOX1	-0.677	0.140	0.935	0.054	0.732	0.098	-0.505	0.307
DOX1^{ON}-D3	0.455	0.365	-0.640	0.171	-0.699	0.123	-0.169	0.750
DOX1^{ON}-D5	-0.468	0.349	-0.860	0.252	-0.354	0.492	-0.865	0.234
DOX1^{ON}-D8	-0.082	0.878	-0.422	0.404	-0.129	0.808	-0.306	0.555
DOX^{OFF}	0.613	0.196	-0.326	0.529	-0.668	0.147	0.389	0.446
Pre-DOX2	-0.367	0.475	0.273	0.601	0.630	0.180	0.010	0.985
DOX2^{ON}-D3	0.001	0.999	-0.528	0.282	-0.768	0.074	-0.720	0.106
DOX2^{ON}-D5	0.097	0.855	-0.738	0.094	-0.727	0.102	-0.694	0.126
**All phases >2 S.D.**	-0.031	0.862	-0.247	0.158	-0.091	0.609	-0.100	0.573

Supplementary Table 1. Disruption in hindlimb phase did not correlate with speed-related gait parameters. Time point comparisons showed hindlimb phase did not significantly correlate with speed-associated gait measures such as stance, swing, and stride time as well as stride distance. All phase values >2 S.D. also did not significantly correlate with speed-related gait measures (averaged data; Pearson correlation with the Bonferroni correction for multiple comparisons to reduce the likelihood of Type I errors).

Supplementary Table 2.

	Stance time		Swing time		Stride time		Stride length	
	**$r_{y(2 \cdot 1)}$ value**	**p value**	**$r_{y(2 \cdot 1)}$ value**	**p value**	**$r_{y(2 \cdot 1)}$ value**	**p value**	**$r_{y(2 \cdot 1)}$ value**	**p value**
Baseline	0.663	0.223	0.766	0.131	0.862	0.060	0.842	0.073
Pre-DOX1	-0.414	0.323	0.645	0.051	0.306	0.487	0.246	0.582
$DOX1^{ON}$-D3	0.025	0.965	-0.544	0.274	-0.801	0.342	-0.753	0.071
$DOX1^{ON}$-D5	-0.700	0.110	-0.809	0.261	-0.858	0.054	-0.867	**0.027**
$DOX1^{ON}$-D8	-0.961	0.072	-0.463	0.430	-0.669	0.215	-0.809	0.095
DOX^{OFF}	-0.327	0.488	0.014	0.978	-0.284	0.553	-0.267	0.579
Pre-DOX2	0.633	0.206	0.045	0.939	0.599	0.241	0.534	0.312
$DOX2^{ON}$-D3	-0.966	0.072	-0.557	0.329	-0.852	0.067	-0.883	0.423
$DOX2^{ON}$-D5	-0.915	0.243	-0.744	0.146	-0.840	0.072	-0.930	0.180
**All phases >2 S.D.**	-0.039	0.827	-0.258	0.148	-0.140	0.436	-0.162	0.368

Supplementary Table 2. Hindlimb phase versus gait after controlling for speed. Part correlations were performed, where the relationship between phase and gait (e.g., stance time) was measured after controlling for the effect of speed on that gait variable. Hindlimb phase significantly correlated with stride distance at $DOX1^{ON}$-D5 only. This represents approximately 2.8% of the total phase versus gait comparisons analyzed. All hindlimb phase values >2 S.D. did not significantly correlate with gait (averaged data; Part correlation with Bonferroni correction for multiple comparisons).

Supplementary Table 3.

		**p value**	**U^2 value**
Baseline vs	Pre-DOX1	$p>0.50$	-0.13369
	DOX1^{ON}-D3	$0.05<p<0.10$	0.16895
	DOX1^{ON}-D5	**$0.002<p<0.005$**	0.33339
	DOX1^{ON}-D8	$0.10<p<0.20$	0.13157
	All-DOX1^{ON}	**$p<0.001$**	0.56176
	DOX^{OFF}	$p>0.5$	0.07047
Pre-DOX1 vs	DOX1^{ON}-D3	$0.20<p<0.50$	0.08912
	DOX1^{ON}-D5	**$0.01<p<0.02$**	0.24507
	DOX1^{ON}-D8	**$0.02<p<0.05$**	0.21514
	All-DOX1^{ON}	**$p<0.001$**	0.59762
	DOX^{OFF}	$p>0.50$	0.00498
	Pre-DOX2	$p>0.50$	0.01499
Baseline + Pre-DOX1 vs	All-DOX1^{ON}	**$p<0.001$**	0.59762
Pre-DOX2 vs	DOX^{OFF}	$p>0.50$	0.07400
	DOX2^{ON}-D3	**$0.002<p<0.005$**	0.31224
	DOX2^{ON}-D5	**$p<0.001$**	0.39267
	All-DOX2^{ON}	**$p<0.001$**	1.29965

Supplementary Table 3. Silencing L2-L5 interneurons functionally uncouples the left and right hindlimbs during overground stepping. Using the non-parametric two-sample U^2 test, we tested the null hypothesis that the two samples (e.g. Baseline vs DOX1^{ON}-D5) came from two populations with the same directions (in other words, degree of concentration or dispersion). This is an indication of whether the limbs are coupled (phases concentrated in same direction) or uncoupled (phases are dispersed). Silencing the L2-L5 interneurons significantly decreased the concentration of the phase values (reduced clustering at 0.5) and caused an increased dispersion throughout the coordination range. This suggests the hindlimbs became functionally uncoupled during overground locomotion. (Critical value of Watson's $U^2_{(0.05,\infty,\infty)} = 0.1869$; Appendix D, Table D.44)[20].

Supplementary Table 4.

			**p value**	**U^2 value**
Forelimb stepping	Baseline vs	Pre-DOX1	p>0.50	0.01735
		$DOX1^{ON}$-D3	p>0.50	0.03821
		$DOX1^{ON}$-D5	p>0.50	-0.03716
		$DOX1^{ON}$-D8	p>0.50	0.06999
		All-$DOX1^{ON}$	p>0.50	0.00771
		DOX^{OFF}	p>0.50	0.02585
	Pre-DOX1 vs	$DOX1^{ON}$-D3	p>0.50	0.02560
		$DOX1^{ON}$-D5	p>0.50	0.03907
		$DOX1^{ON}$-D8	p>0.50	0.02254
		All-$DOX1^{ON}$	p>0.50	0.07072
		DOX^{OFF}	p>0.50	0.03725
Hindlimb swimming	Baseline vs	Pre-DOX1	p>0.50	0.02585
		$DOX1^{ON}$-D3	p>0.50	-0.96664
		$DOX1^{ON}$-D5	p>0.50	-0.54443
		$DOX1^{ON}$-D8	p>0.50	-0.48521
		All-$DOX1^{ON}$	p>0.50	-1.62815
		DOX^{OFF}	**0.02<p<0.05**	0.20163
	Pre-DOX1 vs	$DOX1^{ON}$-D3	p>0.50	-1.3616
		$DOX1^{ON}$-D5	p>0.50	-0.79496
		$DOX1^{ON}$-D8	p>0.50	-0.92183
		All-$DOX1^{ON}$	p>0.50	-2.69285
		DOX^{OFF}	p>0.50	0.07581

Supplementary Table 4. The conditional silencing of L2-L5 interneurons does not uncouple the forelimbs during stepping nor the hindlimbs during swimming. Following methods described above, the null hypothesis was not rejected for time point comparisons of forelimb stepping and hindlimb swimming. Silencing the L2-L5 interneurons did not change the concentration of the phase values at 0.5. Note that in hindlimb swimming, Baseline was significantly different from DOX^{OFF} wherein the phases were more clustered at 0.5. (Critical value of Watson's $U^2_{(0.05,\infty,\infty)} = 0.1869$; Appendix D, Table D.44)[20].
