# Supplementary material for: Reversible silencing of lumbar spinal interneurons unmasks a task-specific network for securing hindlimb alternation
Source: Nat Commun. 2017 Dec 6;8:1963. doi: 10.1038/s41467-017-02033-x (PMC5719045; doi:10.1038/s41467-017-02033-x)
Supplement: Supplementary file 3 — Description of Additional Supplementary Files [file 41467_2017_2033_MOESM3_ESM.pdf]

Description of Additional Supplementary Files

File Name: Supplementary Movie 1
Description: Silencing L2-L5 interneurons disrupts hindlimb alternation during stepping. Prior to silencing, the hindlimbs alternated during overground stepping. DOXON-induced silencing disrupted alternation, including bouts of hindlimb “hopping.”

File Name: Supplementary Movie 1
Description: Hindlimb alternation persists during swimming when L2-L5 interneurons are conditionally silenced. Silencing L2-L5 interneurons did not disrupt hindlimb alternation during swimming.
